# Exploring the Usefulness of Tumor Budding as a Histopathological Marker Compared to Tumor-Node-Metastasis (TNM) Staging in Breast Cancer

**DOI:** 10.7759/cureus.70315

**Published:** 2024-09-27

**Authors:** Soumya Agrawal, Sunita Vagha

**Affiliations:** 1 Department of Pathology, Jawaharlal Nehru Medical College, Datta Meghe Institute of Higher Education and Research, Wardha, IND

**Keywords:** breast cancer, histological grade, prognostic markers, tnm staging, tumor budding

## Abstract

Background: Tumor budding, defined as small clusters of tumor cells at the invasive front of carcinomas, has gained attention as a potential prognostic marker in various cancers. This study aimed to evaluate the utility of tumor budding as a histopathological marker in breast cancer and compare it to traditional prognostic markers such as histological grading, tumor-node-metastasis (TNM) staging, and molecular subtypes.

Methods: A prospective, cross-sectional study was conducted over two years (June 2022 to May 2024) in the Department of Pathology at Jawaharlal Nehru Medical College, Wardha. Seventy-two female patients diagnosed with breast carcinoma who underwent modified radical mastectomy were included. Tumor budding was assessed through histopathological analysis and categorized as high or low. Statistical correlations were established between tumor budding and tumor size, histological grade, lymph node involvement, vascular invasion, and molecular subtypes (estrogen receptor, progesterone receptor, human epidermal growth factor receptor 2, and triple-negative breast cancer, TNBC). The chi-square test and multiple regression analyses were applied to assess significance.

Results: High tumor budding was observed in 68.1% of patients and was significantly associated with larger tumor size (p = 0.040), higher histological grade (p = 0.009), lymph node metastasis (p = 0.002), and vascular invasion (p = 0.003). The postmenopausal age group (>55 years) demonstrated a higher prevalence of high budding (p = 0.010). No significant correlation was found between tumor budding and molecular subtypes (p = 0.39), although high budding was more frequent in TNBC cases.

Conclusion: Tumor budding is significantly associated with more aggressive tumor characteristics in breast cancer. Incorporating tumor budding into routine pathological assessments alongside TNM staging and histological grading may enhance the ability to identify high-risk patients and guide treatment strategies. Further large-scale, multicenter studies are warranted to confirm its prognostic value.

## Introduction

Breast cancer remains the leading cause of cancer-related deaths among women worldwide, with an estimated 2.3 million new cases diagnosed in 2020 alone [1]. It accounts for approximately 25% of all cancers in women, making it a significant public health challenge [2]. Advances in early detection, such as mammography and targeted therapies, have improved survival rates; however, many patients still face challenges related to tumor recurrence, metastasis, and resistance to treatment [3]. Therefore, there is an urgent need for reliable prognostic markers that can predict disease aggressiveness and guide individualized treatment strategies. Traditionally, prognostic markers for breast cancer include histological grading, tumor-node-metastasis (TNM) staging, and molecular profiling, such as the expression of estrogen receptor (ER), progesterone receptor (PR), and human epidermal growth factor receptor 2 (HER2). These markers provide essential information about tumor biology and guide clinical decisions regarding treatment [4]. However, breast cancer is a highly heterogeneous disease, and even within the same TNM stage or molecular subtype, patient outcomes can vary significantly [5]. This variability underscores the need for additional markers to predict the likelihood of recurrence, metastasis, and overall survival with greater precision.

One such emerging market is tumor budding, which refers to the presence of isolated single cells or small clusters of cells (fewer than five) at the invasive front of the tumor [6]. Initially described in colorectal cancer, tumor budding has gained recognition as a powerful prognostic marker linked to poor outcomes, including metastasis and overall survival [7]. Studies in colorectal cancer have demonstrated that high tumor budding correlates with lymph node metastasis, advanced disease stages, and reduced survival [8]. Due to these findings, tumor budding has been incorporated into clinical practice guidelines for colorectal cancer [9]. In recent years, interest in the role of tumor budding in other cancers, including breast cancer, has increased. Tumor budding in breast cancer has been associated with aggressive tumor characteristics, such as high histological grade, larger tumor size, lymphovascular invasion, and lymph node metastasis [10]. Some studies suggest that tumor budding may serve as an independent prognostic factor, particularly in triple-negative breast cancer (TNBC), a subtype known for its poor prognosis and limited treatment options [11]. The presence of tumor budding in TNBC could indicate a more invasive phenotype, contributing to the tumor's ability to spread and evade treatment [12].

Despite these promising findings, the role of tumor budding in breast cancer is not yet fully understood. Most of the research has been conducted in small cohorts, and its prognostic value relative to established markers such as TNM staging and molecular subtypes remains under investigation [13]. Moreover, the methods used to assess tumor budding can vary, leading to potential discrepancies in the reported significance of this marker [14]. Standardizing the criteria for tumor budding assessment is critical for its broader adoption in clinical practice. Given the growing body of evidence supporting the prognostic significance of tumor budding in various cancers, this study aims to evaluate the utility of tumor budding as a histopathological marker in breast cancer. By comparing tumor budding with traditional prognostic markers such as histological grade, TNM staging, and molecular subtypes, this study seeks to determine whether tumor budding can provide additional prognostic information and improve the stratification of breast cancer patients for treatment.

## Materials and methods

Study design and setting

This observational, cross-sectional, and prospective study was conducted over two years, from June 2022 to May 2024. The study occurred in the Histopathology and Immunohistochemistry Division of the Department of Pathology, Jawaharlal Nehru Medical College, Wardha, in coordination with the Department of General Surgery at Acharya Vinoba Bhave Rural Hospital, Wardha. The Institutional Ethics Committee approved the study protocol, and written informed consent was obtained from all participants.

Study population and sample size

A total of 72 female patients diagnosed with breast carcinoma and who underwent modified radical mastectomy (MRM) were included in the study. The sample size was calculated using a formula based on the incidence of breast carcinoma in women (13.5%) and a desired margin of error of 8%, resulting in a required sample size of 70. However, 72 patients were ultimately recruited for the study. Patients were selected based on specific inclusion and exclusion criteria.

Inclusion and exclusion criteria

Patients were included if they had histopathologically confirmed breast carcinoma, newly diagnosed cases, and specimens from MRMs. Exclusion criteria included patients diagnosed with other lesions (such as myoepithelial or mesenchymal tumors), those with a history of lobectomy or biopsy (including Tru-cut and wedge biopsies), male breast cancer patients, and individuals who had undergone neoadjuvant therapy.

Specimen collection and processing

MRM specimens from confirmed cases of breast carcinoma were collected at the Department of General Surgery and subsequently transferred to the Department of Pathology. Specimens were fixed in 10% formalin for a maximum of 24 hours. The specimens were grossed by slicing at 1 cm intervals, starting from the nipple-areola complex toward the lateral skin margin. Sections were taken from tumor masses, surgical margins (superior, inferior, medial, and lateral), tumor bases, nipple-areola complexes, and normal breast areas. Axillary lymph nodes were also sectioned and examined.

Histopathological and immunohistochemical analysis

Tissue sections were processed using automated tissue processors (Leica TP 1020, Leica Biosystems, Wetzlar, Germany) [15] and sectioned into 5-micron thick slices using a rotary microtome (Leica RM 2125 RT, Leica Microsystems, Wetzlar, Germany) [16]. Hematoxylin and eosin [17]. Staining was performed for initial examination and diagnosis. Immunohistochemical staining was done for ER, PR, HER2/neu, and Ki67, using specific antibodies (Pathological Nodal Stage ITC2). CD34 immunostaining was performed on tumor tissues. The slides were evaluated for histological grading based on the Scarff-Bloom-Richardson system [18].

Ethical considerations

Ethical approval was obtained from the Institutional Ethics Committee of Jawaharlal Nehru Medical College, Wardha, before the commencement of the study. Informed consent was obtained from all participants after thoroughly explaining the study's purpose, procedures, potential risks, and benefits. Participants were assured of their right to withdraw from the study at any point without any consequences to their medical care. Confidentiality and anonymity of all patient data were maintained throughout the study, ensuring that personal identifiers were removed from the collected data. All data were securely stored and accessible only to the study team.

Statistical analysis

Statistical analysis was performed using the chi-square test to assess the relationships between tumor budding, histopathological indices, and immunohistochemical markers. Multiple linear regression analysis was applied to determine the factors associated with metastasis. A p value of <0.05 was considered statistically significant.

## Results

Table 1 shows that the patient cohort was nearly evenly distributed across premenopausal, perimenopausal, and postmenopausal age groups. The postmenopausal group, however, had the highest representation at 38.89%. Both premenopausal and perimenopausal groups accounted for 30.56% each. The mean age of the patients was 53.61 years, with a range spanning from 26 to 85 years.

**Table 1:** Distribution of cases according to age

Age group (years)	No. of patients (n)	Percentage (%)
Premenopausal (<48 years)	22	30.56
Perimenopausal (48-55 years)	22	30.56
Postmenopausal (>55 years)	28	38.89
Total	72	100

Table 2 shows that a significant percentage of patients (59.7%) had tumors ranging from 2 to 5 cm in size (T2 stage). A smaller number had tumors larger than 5 cm (T3, 13.9%) or involving the chest wall (T4, 16.7%), while only 9.7% had tumors measuring up to 2 cm (T1).

**Table 2:** Distribution of cases according to tumor size T1: tumor size up to 2 cm; T2: tumor size between 2 and 5 cm; T3: tumor size larger than 5 cm; T4: tumor involving the chest wall

Tumor size (cm)	No. of patients (n)	Percentage (%)
T1 (up to 2 cm)	7	9.7
T2 (2-5 cm)	43	59.7
T3 (>5 cm)	10	13.9
T4 (involvement of chest wall)	12	16.7
Total	72	100

Table 3 shows that most patients were diagnosed with grade II tumors (51.39%), followed by grade III tumors (33.33%). A smaller proportion had grade I tumors (15.28%), indicating that most patients presented with moderately to poorly differentiated tumors.

**Table 3:** Distribution of cases according to BR on histological grade BR: Bloom-Richardson (a system used for histological grading of breast cancer)

BR grade	No. of patients (n)	Percentage (%)
I	11	15.28
II	37	51.39
III	24	33.33
Total	72	100

Table 4 shows that lymph node involvement was absent (N0) in 45.83% of cases. However, 54.17% of patients had some degree of lymph node metastasis, with 23.61% in the N1 stage, 16.67% in N2, and 11.11% in N3, reflecting a substantial burden of nodal involvement.

**Table 4:** Distribution of cases according to lymph node involvement Nx: regional lymph nodes cannot be assessed; N0: no regional lymph node metastasis; N1: metastasis to 1-3 axillary lymph nodes; N2: metastasis to 4-9 axillary lymph nodes; N3: metastasis to 10 or more axillary lymph nodes

Stage	No. of patients (n)	Percentage (%)
Nx	2	2.78
N0	33	45.83
N1	17	23.61
N2	12	16.67
N3	8	11.11
Total	72	100

Table 5 shows that vascular invasion was noted in 36.11% of cases, while most patients (63.89%) did not exhibit this feature. This suggests that a significant subset of the patient population had aggressive disease characteristics associated with vascular involvement.

**Table 5:** Distribution of cases according to vascular invasion

Vascular invasion	No. of patients (n)	Percentage (%)
Negative	46	63.89
Positive	26	36.11
Total	72	100

Table 6 shows that TNBC was the most prevalent among the molecular subtypes, affecting 36.11% of patients. Luminal A accounted for 29.17%, followed by luminal B at 22.22% and HER2 enriched at 12.50%, indicating a wide distribution of breast cancer subtypes within the cohort.

**Table 6:** Distribution of cases according to immunophenotypes HER2: human epidermal growth factor receptor 2; TNBC: triple-negative breast cancer (tumors lacking estrogen receptor, progesterone receptor, and HER2 expression)

Molecular subtype	No. of patients (n)	Percentage (%)
Luminal A	21	29.17
Luminal B	16	22.22
HER2 enriched	9	12.50
TNBC	26	36.11
Total	72	100

Table 7 shows that tumor budding was categorized as high in 68.1% of the cases, while 31.9% of patients had low tumor budding. This observation suggests that many patients presented with more aggressive tumor characteristics associated with high budding.

**Table 7:** Distribution of cases according to tumor budding

Tumor budding	No. of patients (n)	Percentage (%)
Low	23	31.9
High	49	68.1
Total	72	100

Table 8 shows a significant correlation between age and tumor budding (p = 0.010). High tumor budding was more common in the postmenopausal group (>55 years, 33.33%), while low budding was observed predominantly in the perimenopausal group (48-55 years, 16.67%) and premenopausal patients (<48 years, 9.72%).

**Table 8:** Correlation between tumor budding and age in years ^*^Statistically significant (p value < 0.05)

Age	Low	High	Total	χ^2^ value	p value
<48 years	7 (9.72%)	15 (20.83%)	22 (30.56%)	9.18	0.010^*^
48-55 years	12 (16.67%)	10 (13.89%)	22 (30.56%)		
>55 years	4 (5.56%)	24 (33.33%)	28 (38.89%)		
Total	23 (31.94%)	49 (68.06%)	72 (100%)		

Table 9 shows a significant association (p = 0.040) between tumor budding and size. Patients with larger tumors (T3 and T4) were likelier to have high budding, while those with smaller tumors (T1) tended to exhibit low budding.

**Table 9:** Correlation between tumor budding and tumor size (cm) T1: tumor size up to 2 cm; T2: tumor size between 2 and 5 cm; T3: tumor size larger than 5 cm; T4: tumor involving the chest wall ^*^Statistically significant (p value < 0.05)

Tumor size (cm)	Low	High	Total	χ^2^ value	p value
T1 (up to 2 cm)	2 (2.78%)	5 (6.94%)	7 (9.72%)	8.29	0.040^*^
T2 (2-5 cm)	19 (26.39%)	24 (33.33%)	43 (59.72%)		
T3 (>5 cm)	1 (1.39%)	9 (12.50%)	10 (13.89%)		
T4 (involvement of chest wall)	1 (1.39%)	11 (15.28%)	12 (16.67%)		
Total	23 (31.94%)	49 (68.06%)	72 (100%)		

Table 10 shows a significant correlation between tumor budding and histological grade (p = 0.009). High tumor budding was more frequently observed in patients with grades II and III, while low budding was more common in grade I tumors.

**Table 10:** Correlation between tumor budding and BR grading BR: Bloom-Richardson (a system used for histological grading of breast cancer) ^*^Statistically significant (p value < 0.05)

BR grading	Low	High	Total	χ^2^ value	p value
I	7 (9.72%)	4 (5.56%)	11 (15.28%)	9.42	0.009^*^
II	13 (18.06%)	24 (33.33%)	37 (51.39%)		
III	3 (4.17%)	21 (29.17%)	24 (33.33%)		
Total	23 (31.94%)	49 (68.06%)	72 (100%)		

Table 11 shows a significant association (p = 0.002) between tumor budding and lymph node involvement. High tumor budding was notably more prevalent in patients with advanced nodal involvement (N1, N2, and N3 stages), while low budding was more common in patients without lymph node metastasis (N0).

**Table 11:** Correlation between tumor budding and N-stage N0: no regional lymph node metastasis; N1: metastasis to 1-3 axillary lymph nodes; N2: metastasis to 4-9 axillary lymph nodes; N3: metastasis to 10 or more axillary lymph nodes ^*^Statistically significant (p value < 0.05)

N-stage	Low	High	Total	χ^2^ value	p value
N0	18 (25.71%)	15 (21.43%)	33 (47.14%)	14.71	0.002^*^
N1	2 (2.86%)	15 (21.43%)	17 (24.29%)		
N2	3 (4.29%)	9 (12.86%)	12 (17.14%)		
N3	0 (0%)	8 (11.43%)	8 (11.43%)		
Total	23 (32.86%)	47 (67.14%)	70 (100%)		

Table 12 shows that tumor budding was significantly associated with vascular invasion (p = 0.003). High tumor budding was more frequent in cases with vascular invasion (51.39%), while low budding was more common in patients without vascular invasion (12.50%).

**Table 12:** Correlation between tumor budding and vascular invasion ^*^Statistically significant (p value <0.05)

Vascular invasion	Low	High	Total	χ^2^ value	p value
Negative	9 (12.50%)	37 (51.39%)	46 (63.89%)	8.97	0.003^*^
Positive	14 (19.44%)	12 (16.67%)	26 (36.11%)		
Total	23 (31.94%)	49 (68.06%)	72 (100%)		

Table 13 shows no significant correlation between tumor budding and immunophenotypes (p = 0.39). However, high tumor budding was slightly more common in patients with TNBC (20.83%) and luminal A (19.44%), while low budding was more frequently seen in luminal A cases (9.72%).

**Table 13:** Correlation between tumor budding and immunophenotype HER2: human epidermal growth factor receptor 2; TNBC: triple-negative breast cancer ^*^Not statistically significant

Immunophenotype	Low	High	Total	χ^2^ value	p value
Luminal A	7 (9.72%)	14 (19.44%)	21 (29.17%)	2.97	0.39^*^
Luminal B	3 (4.17%)	13 (18.06%)	16 (22.22%)		
HER2 enriched	2 (2.78%)	7 (9.72%)	9 (12.50%)		
TNBC	11 (15.28%)	15 (20.83%)	26 (36.11%)		
Total	23 (31.94%)	49 (68.06%)	72 (100%)		

## Discussion

The current study highlights the utility of tumor budding as a histopathological marker and its association with traditional prognostic factors, such as TNM staging, histological grade, lymph node involvement, and molecular subtypes, in breast cancer. Tumor budding has gained attention as an important marker of tumor aggressiveness in several cancers, including colorectal and gastric carcinomas [6,19], but its role in breast cancer remains underexplored. Our findings contribute to this growing body of literature by demonstrating significant correlations between tumor budding and more aggressive tumor features in breast cancer patients. Tumor budding, defined by small clusters of tumor cells at the invasive front of carcinomas, is increasingly recognized as a marker for increased invasiveness and metastatic potential [14]. This study observed high tumor budding in 68.1% of the cases. This high frequency suggests that tumor budding may be a prevalent feature in breast carcinoma, particularly in more advanced or aggressive cases. The association between high tumor budding and increased tumor size (T3 and T4 stages) is in line with the hypothesis that tumors with higher budding tend to be larger and more invasive [20]. Larger tumors are often associated with greater tumor heterogeneity, and tumor budding may represent one aspect of this complexity, reflecting the tumor's ability to invade surrounding tissues and metastasis [21].

Histological grading, particularly the Scarff-Bloom-Richardson system, remains a cornerstone in breast cancer prognosis, and its integration with tumor budding presents an interesting possibility. Our study strongly associated high tumor budding with higher histological grades (grades II and III). This finding is consistent with previous research in other cancers, where high tumor budding was more common in poorly differentiated tumors [22,23]. Poor differentiation implies that the tumor cells have lost their normal characteristics and function, which is associated with more aggressive behavior. Tumor budding, as a histological marker of this dedifferentiation process, further emphasizes the aggressive nature of these tumors. The correlation between tumor budding and higher histological grades could enhance the prognostic value of histological grading, providing pathologists and oncologists with an additional marker to evaluate tumor aggressiveness. One of the key findings of this study is the strong association between tumor budding and lymph node involvement. Over half of the patients in the study (54.17%) exhibited lymph node metastasis, and those with high tumor budding were significantly more likely to have advanced nodal involvement (N1, N2, and N3 stages). This supports the notion that tumor budding is a reflection of the tumor's capacity to spread through the lymphatic system, which is a well-established pathway for metastasis in breast cancer [24]. Tumor budding likely represents an early event in the epithelial-mesenchymal transition (EMT), a process that enables tumor cells to lose their epithelial characteristics and gain mesenchymal traits, allowing them to detach from the primary tumor mass and invade adjacent tissues [25]. The link between EMT and tumor budding has been studied extensively, and our findings are consistent with the literature that positions budding as a key step in metastasis [13]. This makes tumor budding a particularly valuable marker for predicting lymph node metastasis, which is one of the most critical factors in breast cancer prognosis and treatment planning.

Another important factor evaluated in this study is vascular invasion, a hallmark of aggressive tumor behavior. Tumor cells that invade blood vessels are more likely to disseminate hematogenously, leading to distant metastasis. In our cohort, high tumor budding was significantly associated with vascular invasion, indicating that budding tumors are more capable of entering the vascular system [8]. This finding adds to the growing evidence that tumor budding is a marker for local invasiveness and a predictor of systemic dissemination. Previous studies in colorectal cancer have shown similar associations, where high tumor budding was linked with both lymphatic invasion and vascular invasion, emphasizing its role in the metastatic process [26]. Therefore, incorporating tumor budding into routine pathological assessment could provide valuable information on a tumor's potential to metastasize locally and distantly. Interestingly, we found no statistically significant association between tumor budding and molecular subtypes of breast cancer (p = 0.39), although high tumor budding was more frequent in TNBC. This result suggests that tumor budding may operate independently of the tumor's molecular profile, acting as a universal marker of aggressiveness regardless of subtype. While TNBC is typically considered one of the most aggressive subtypes due to its lack of hormone receptors and HER2 expression [27], the absence of a significant correlation between budding and molecular markers may indicate that tumor budding represents a distinct pathological feature that transcends molecular classifications. This aligns with the notion that tumor budding is a histopathological marker of the tumor's invasive capacity rather than its molecular or genetic signature [28]. However, given the small sample size in this study, further research with larger cohorts is needed to explore whether tumor budding may have different implications in various molecular subtypes.

The findings of this study should be considered in light of certain limitations. First, the sample size of 72 patients is relatively small, and the study was conducted at a single institution, which may limit the generalizability of the results. Additionally, excluding patients who underwent neoadjuvant therapy limits the ability to assess the role of tumor budding in patients who receive such treatment before surgery. Neoadjuvant therapy can impact tumor morphology, and further research is needed to determine how tumor budding behaves in treated tumors. Another limitation is the cross-sectional nature of this study, which does not allow for long-term follow-up to evaluate the impact of tumor budding on patient outcomes such as survival and recurrence. Future studies should incorporate longitudinal data to better understand the prognostic significance of tumor budding over time. Moreover, while our study found significant correlations between tumor budding and several aggressive features, interobserver variability in the assessment of tumor budding remains a challenge. Standardized criteria and training for pathologists are necessary to ensure consistent and reproducible evaluations across different centers [29].

Limitations

A key limitation of this study is the relatively small sample size of 72 patients, which may limit the generalizability of the findings to the broader breast cancer population. Additionally, the study was conducted at a single institution, which may introduce selection bias and limit the diversity of the patient cohort. Another limitation is the exclusion of patients who received neoadjuvant therapy, which prevents the evaluation of tumor budding in this specific subset of breast cancer patients. Furthermore, while tumor budding was assessed using established histopathological techniques, interobserver variability in the evaluation of budding could affect the consistency and reproducibility of the results. Finally, the study's cross-sectional design restricts the ability to assess the prognostic impact of tumor budding over time, as longitudinal follow-up data were not included to correlate budding with clinical outcomes such as recurrence and survival. These limitations suggest that further research, including larger, multicenter studies with long-term follow-up, is necessary to validate the role of tumor budding as a prognostic marker in breast cancer.

## Conclusions

In conclusion, this study underscores the potential of tumor budding as a valuable histopathological marker in breast cancer prognosis. The significant correlations between high tumor budding and factors such as larger tumor size, higher histological grade, lymph node involvement, and vascular invasion highlight its association with more aggressive tumor characteristics. Although no significant relationship was found with molecular subtypes, the prevalence of high budding in TNBC suggests its relevance in identifying high-risk cases. Incorporating tumor budding into routine pathological assessments alongside traditional markers like TNM staging and histological grading may enhance the accuracy of prognostication and guide treatment decisions, ultimately improving patient outcomes in breast cancer management.

## References

[1] Bray F, Ferlay J, Soerjomataram I, Siegel RL, Torre LA, Jemal A (2018). Global cancer statistics 2018: GLOBOCAN estimates of incidence and mortality worldwide for 36 cancers in 185 countries. CA Cancer J Clin.

[2] Siegel RL, Miller KD, Jemal A (2020). Cancer statistics, 2020. CA Cancer J Clin.

[3] Waks AG, Winer EP (2019). Breast cancer treatment: a review. JAMA.

[4] Rakha EA, Reis-Filho JS, Baehner F (2010). Breast cancer prognostic classification in the molecular era: the role of histological grade. Breast Cancer Res.

[5] Polyak K (2011). Heterogeneity in breast cancer. J Clin Invest.

[6] Lugli A, Karamitopoulou E, Zlobec I (2012). Tumour budding: a promising parameter in colorectal cancer. Br J Cancer.

[7] Zlobec I, Lugli A (2018). Tumour budding in colorectal cancer: molecular rationale for clinical translation. Nat Rev Cancer.

[8] Grigore AD, Jolly MK, Jia D, Farach-Carson MC, Levine H (2016). Tumor budding: the name is EMT. Partial EMT. J Clin Med.

[9] De Smedt L, Palmans S, Sagaert X (2016). Tumour budding in colorectal cancer: what do we know and what can we do?. Virchows Arch.

[10] Huang T, Bao H, Meng YH, Zhu JL, Chu XD, Chu XL, Pan JH (2022). Tumour budding is a novel marker in breast cancer: the clinical application and future prospects. Ann Med.

[11] Salhia B, Trippel M, Pfaltz K (2015). High tumor budding stratifies breast cancer with metastatic properties. Breast Cancer Res Treat.

[12] Cserni G, Quinn CM, Foschini MP (2021). Triple-negative breast cancer histological subtypes with a favourable prognosis. Cancers (Basel).

[13] van Wyk HC, Park J, Roxburgh C, Horgan P, Foulis A, McMillan DC (2015). The role of tumour budding in predicting survival in patients with primary operable colorectal cancer: a systematic review. Cancer Treat Rev.

[14] Koelzer VH, Langer R, Zlobec I, Lugli A (2014). Tumor budding in upper gastrointestinal carcinomas. Front Oncol.

[15] (2024). Leica TP1020 | Automatic benchtop tissue processor, semi-enclosed. https://www.leicabiosystems.com/en-in/histology-equipment/tissue-processors/leica-tp1020/.

[16] (2024). Leica RM2125 RTS | Rotary microtome for histology. https://www.leicabiosystems.com/en-in/histology-equipment/microtomes/leica-rm2125-rts/.

[17] (2024). An intro to H&E staining: protocol, best practices, steps & more. https://www.leicabiosystems.com/en-in/knowledge-pathway/he-staining-overview-a-guide-to-best-practices/.

[18] (2024). Staging & grade - breast pathology | Johns Hopkins Pathology. https://pathology.jhu.edu/breast/staging-grade/.

[19] Cho SJ, Kakar S (2018). Tumor budding in colorectal carcinoma: translating a morphologic score into clinically meaningful results. Arch Pathol Lab Med.

[20] Kanazawa H, Mitomi H, Nishiyama Y, Kishimoto I, Fukui N, Nakamura T, Watanabe M (2008). Tumour budding at invasive margins and outcome in colorectal cancer. Colorectal Dis.

[21] Rakha EA, El-Sayed ME, Green AR, Lee AH, Robertson JF, Ellis IO (2007). Prognostic markers in triple-negative breast cancer. Cancer.

[22] Xiao SM, Li J (2023). Tumor budding in gastric cancer. World J Gastrointest Surg.

[23] Saito K, Okuyama T, Miyazaki S (2022). Tumor budding as a predictive marker of relapse and survival in patients with stage II colon cancer. In Vivo.

[24] Ueno H, Murphy J, Jass JR, Mochizuki H, Talbot IC (2002). Tumour 'budding' as an index to estimate the potential of aggressiveness in rectal cancer. Histopathology.

[25] Mitrovic B, Schaeffer DF, Riddell RH, Kirsch R (2012). Tumor budding in colorectal carcinoma: time to take notice. Mod Pathol.

[26] Micalizzi DS, Farabaugh SM, Ford HL (2010). Epithelial-mesenchymal transition in cancer: parallels between normal development and tumor progression. J Mammary Gland Biol Neoplasia.

[27] Zlobec I, Lugli A (2008). Prognostic and predictive factors in colorectal cancer. Postgrad Med J.

[28] Rakha EA, Reis-Filho JS, Ellis IO (2008). Basal-like breast cancer: a critical review. J Clin Oncol.

[29] Ueno H, Mochizuki H, Hashiguchi Y (2008). Histological grading of colorectal cancer: a simple and objective method. Ann Surg.

